# Using linked administrative data to aid the handling of non-response and restore sample representativeness in cohort studies: the 1958 national child development study and hospital episode statistics data

**DOI:** 10.1186/s12874-023-02099-w

**Published:** 2023-11-11

**Authors:** Nasir Rajah, Lisa Calderwood, Bianca L De Stavola, Katie Harron, George B Ploubidis, Richard J Silverwood

**Affiliations:** 1 Centre for Longitudinal Studies, UCL Social Research Institute, University College London, 20 Bedford Way, London, WC1H 0AL UK; 2 Population, Policy & Practice Research and Teaching Department, UCL Great Ormond Street Institute of Child Health, University College London, 30 Guilford Street, London, WC1N 1EH UK

**Keywords:** Administrative data, Cohort studies, Data linkage, Missing data, Multiple imputation, Representativeness

## Abstract

**Background:**

There is growing interest in whether linked administrative data have the potential to aid analyses subject to missing data in cohort studies.

**Methods:**

Using linked 1958 National Child Development Study (NCDS; British cohort born in 1958, n = 18,558) and Hospital Episode Statistics (HES) data, we applied a LASSO variable selection approach to identify HES variables which are predictive of non-response at the age 55 sweep of NCDS. We then included these variables as auxiliary variables in multiple imputation (MI) analyses to explore the extent to which they helped restore sample representativeness of the respondents together with the imputed non-respondents in terms of early life variables (father’s social class at birth, cognitive ability at age 7) and relative to external population benchmarks (educational qualifications and marital status at age 55).

**Results:**

We identified 10 HES variables that were predictive of non-response at age 55 in NCDS. For example, cohort members who had been treated for adult mental illness had more than 70% greater odds of bring non-respondents (odds ratio 1.73; 95% confidence interval 1.17, 2.51). Inclusion of these HES variables in MI analyses only helped to restore sample representativeness to a limited extent. Furthermore, there was essentially no additional gain in sample representativeness relative to analyses using only previously identified survey predictors of non-response (i.e. NCDS rather than HES variables).

**Conclusions:**

Inclusion of HES variables only aided missing data handling in NCDS to a limited extent. However, these findings may not generalise to other analyses, cohorts or linked administrative datasets. This work provides a demonstration of the use of linked administrative data for the handling of missing cohort data which we hope will act as template for others.

**Supplementary Information:**

The online version contains supplementary material available at 10.1186/s12874-023-02099-w.

## Background

Sample attrition in longitudinal surveys can lead to bias if the remaining respondents are not representative of the survey’s target population. Such selective response is likely to be the norm rather than the exception [1, 2], so appropriate handling of missing data due to attrition (or non-response more generally) is imperative.

Recent decades have seen the establishment of a number of principled methods for the handling of missing data, such as multiple imputation (MI) [3], full information maximum likelihood (FIML) [4] and inverse probability weighting (IPW) [5]. Typically, application of such methods relies on an assumption of “missingness at random” (MAR). MAR implies that given the observed values, missingness does not depend on unobserved values or, equivalently, that systematic differences between the missing values and the observed values can be explained by observed data [6]. Strategies for reducing bias due to non-response may therefore seek to maximise the plausibility of the MAR assumption. This can be achieved by the inclusion of carefully selected auxiliary variables (variables not of direct substantive interest), either in the imputation phase of MI, directly in FIML analysis, or in the derivation of response weights for IPW. Relevant auxiliary variables are those associated with the underlying values of the variable(s) subject to missingness, particularly those also associated with the probability of missingness [3]. An important part of analysing data subject to missingness is often therefore the identification of suitable auxiliary variables.

Variables associated with the underlying values of the variable(s) subject to missingness will generally need to be considered on an analysis-specific basis due to the inclusion of different variables in analytic models. However, since the major driver of missingness in longitudinal surveys will generally be wave (as opposed to item) non-response, variables associated with the probability of missingness can be considered more generically by identifying variables predictive of wave non-response. Analysts can then select variables (assumed to be) associated with the underlying values of the variable(s) subject to missingness from the pool of identified predictors of non-response to include as auxiliary variables. Such predictors of non-response can be identified from within the (often vast) pool of variables previously collected as part of the longitudinal survey.

In recent years, many longitudinal surveys have begun to link administrative records (for example, health, education or financial) for their participants with their data collected as part of the survey. Such linked administrative data often contain broader or more detailed information than conventional survey data and may be more complete, since administrative data typically have the benefit of minimal attrition over time [7]. There is therefore substantial interest in whether variables derived from linked administrative data may be helpful as auxiliary variables in analyses of survey data subject to missingness.

In this paper we explore this idea using data from the 1958 National Child Development Study (NCDS), a long-running British birth cohort [8], for which linkage to secondary care data from the Hospital Episode Statistics (HES) database is available [9–11]. Previous work in NCDS considering only variables collected as part of the study (i.e. not from linked administrative data) found disadvantaged socio-economic background in childhood, worse mental health and lower cognitive ability in early life, and lack of civic and social participation in adulthood to be consistently associated with non-response [12]. The main aim of this paper is to explore whether administrative data have the potential to enhance approaches to handling missingness data in cohort studies – a question which has received recent interest in relation to NCDS [13, 14]. A further aim is that by providing a demonstration of the use of linked administrative data for the handling of missing cohort data our work will act as template for others.

## Methods

### Data

#### 1958 National child development study (NCDS)

The NCDS follows the lives of 18,558 people born in Great Britain in a single week of 1958 [8]. Since the birth sweep, NCDS cohort members have been followed up 10 times, with the eleventh sweep currently underway with the cohort members now aged 64. The study includes information on cohort members’ physical and educational development, economic circumstances, employment, family life, health behaviours, wellbeing, social participation, biological data and attitudes. Although response rates in recent sweeps of NCDS remain relatively high considering the decades-long duration of the study, non-response is a sufficient issue to require careful handling. For example, of the 15,613 NCDS cohort members remaining in the target population (still alive and living in Great Britain) at wave 9 (2013, age 55), 9,137 (58.5%) responded and 6,476 (41.5%) were not observed for one of a number of reasons (refusal, the survey team not been being able to establish contact, or because contact was not attempted, for example because of long-term refusal). Item non-response among respondents is of a relatively lower level, typically less than 10% [15].

#### Hospital episode statistics (HES)

HES is a collection of databases containing details of all admissions (Admitted Patient Care (APC) and Critical Care (CC)), Accident and Emergency (A&E) attendances and Outpatient (OP) appointments at NHS hospitals in England, maintained by NHS Digital [9]. Each HES dataset provides detailed information on admission and discharge or appointment dates, diagnoses, procedures, basic patient demographics, and hospital characteristics [16]. The period of data availability differs by dataset, from 1997 for APC, from 2007 for A&E, from 2009 for CC and from 2003 for OP.

#### Linked NCDS-HES data

Linkage between NCDS and all four HES datasets has recently been undertaken, on the basis of consents obtained at NCDS wave 8 (2008, age 50) [10, 11]. Matching was carried out in two stages: in the first, NHS Digital used information provided by the NCDS team on the cohort members’ name, sex, date of birth and postcode to identify their NHS number; in the second, NHS Digital used the identified NHS number to extract HES data for each cohort member, with pseudo-anonymised linked HES data returned. Because HES data relate to NHS hospitals in England only, we restricted our attention to NCDS cohort members who we considered eligible for HES linkage due to having lived in England for at least one wave been wave 6 (2000, age 42) and wave 9 (2013, age 55) (the period corresponding to HES data availability). The flow of data, from the full sample of NCDS cohort members to the linked samples for each HES dataset, is shown in the data flow diagram in Supplementary Fig. S1. Recent analyses suggest the linkage quality of the NCDS-HES data to be high and the linked sample to retain a good level of population representativeness [17].

**Fig. 1:**
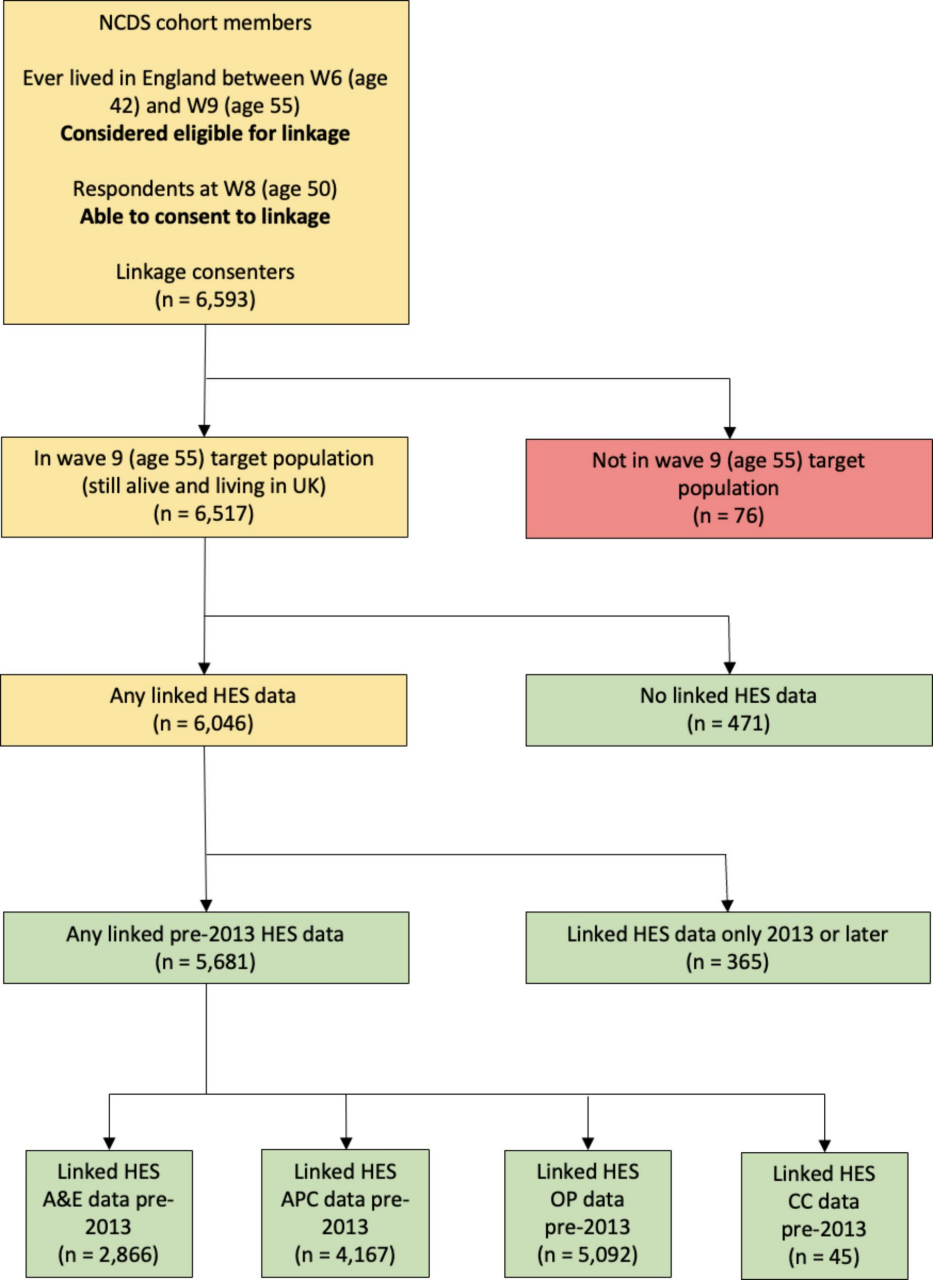
Flow diagram showing 1958 British National Child Development Study-Hospital Episode Statistics data linkage and data availability. APC: admitted patient care; CC: critical care; A&E: accident and emergency; OP: outpatients

In this study we restricted our attention to cohort members who were in the wave 9 (2013, age 55) target population (those who were alive and still living in Great Britain at this point). Individuals outside the target population would not have been in the issued sample for the wave 9 follow-up and therefore could not have responded. As our aim was to identify predictors of non-response and not of mortality or emigration, such individuals were excluded rather than being considered as non-respondents. We used linked HES data from the earliest available date until the end of 2012 to ensure that we only used HES information which pre-dated the point at which response was sought. The impact of these additional criteria on the sample is shown in the data flow diagram in Fig. 1.

#### Annual population survey (APS)

The Annual Population Survey (APS) is a large survey administered yearly by the Office for National Statistics (ONS) [18]. It contains approximately 320,000 respondents and covers social and economic aspects of individuals’ lives. In this study, we used the APS January-December 2013 survey [19] to derive population estimates for the variables of interest, limiting our analysis to 55-year-olds.

### Variables

#### NCDS

In the present analysis we focus on NCDS non-response at wave 9 (age 55). This was captured as a binary variable, defined as cohort members who did not take part in the survey, either because of refusal, the survey team not been being able to establish contact, or because contact was not attempted, for example because of long-term refusal.

Predictors of age 55 NCDS non-response, listed in Supplementary Table S1, were previously identified using survey data from the 10 preceding sweeps (birth to age 50) of NCDS [12].

To assess how effective the identified HES predictors of NCDS age 55 non-response were at restoring sample representativeness despite selective attrition, we considered representativeness with respect to two NCDS variables observed in early life and two NCDS variables in observed in later life (subsequently collectively referred to as “analysis variables”): father’s social class at birth (binary variable for father being in the professional social class), cognitive ability at age 7 (continuous principal component analysis score derived using the scores from the problem arithmetic test, copying designs test, drawing a man test and Southgate Group Reading Test), educational qualifications at age 55 (binary variable for no educational qualifications), and marital status at age 55 (binary variable for single and never married).

#### Linked NCDS-HES data

A total of 58 variables to be considered as potential predictors of NCDS non-response at age 55 were derived across the APC, OP and A&E HES datasets. We aimed to derive as many variables as we could using the information available, though intentionally avoided variables with low sample prevalence which would be unlikely to prove useful as auxiliary variables. We therefore derived variables relating to diagnoses and treatments at a high level (e.g. International Classification of Diseases (ICD)-10 chapters) rather than considering more granular coding. The derived variables relate to the numbers of admissions and appointments, missed appointments, investigations undertaken, diagnoses and treatments received (full details in Supplementary Table S2).

#### APS

For 55-year-olds in APS, we derived the percentage of individuals who were single and had never been married and the percentage of individuals with no educational qualifications using survey information weighted to the mid-2013 population estimate using the weights provided by the ONS [19].

### Statistical analysis

#### HES predictors of NCDS non-response at wave 9 (age 55)

In order to identify which of the 58 derived HES variables were important predictors of non-response at age 55 in NCDS, we employed the least absolute shrinkage and selection operator (LASSO) [20]. We included all 58 HES variables in a logistic regression model for non-response and used the LASSO lambda value that minimised mean cross-validated error using 10-fold cross-validation.

In a secondary analysis we used a multi-stage P value-based variable selection approach, similar to that employed by Mostafa et al. [12], for comparison with the primary approach using the LASSO (see Supplementary Methods S1).

#### Restoring sample representativeness

We undertook several analyses to assess how effective the identified HES predictors of NCDS age 55 non-response were at restoring sample representativeness despite selective attrition. The basic idea underlying each analysis is the same: comparison of a statistic calculated when using data from wave 9 respondents only (so subject to non-response bias) and the same statistic estimated using predictors of non-response as auxiliary variables in MI analyses (to make estimates for the broader sample, including non-respondents) to a known benchmark value. Full details of the analyses are provided in Supplementary Methods S2 but are briefly summarised here.

We first explored the associations between the analysis variables of interest and the identified HES and survey predictors of non-response. This allowed us to assess whether the HES/survey predictors of non-response were sufficiently well associated with the analysis variables to constitute potentially useful auxiliary variables. Associations were explored using linear or logistic regression (as appropriate), with P values from Wald tests of the parameter(s) presented to summarise the strength of evidence for each association.

The first restoring sample representativeness analysis (“Analysis A”) focused on HES linkage consenters who were eligible for linkage and who were within the wave 9 target population. These individuals are non-missing for all HES variables since we assumed those with no linked HES record truly had no relevant hospital interactions. These analyses considered sample representativeness in terms of variables observed in early life (father’s social class at birth and cognitive ability at age 7). The percentage of fathers in professional social class at birth and mean cognitive ability at age 7 were calculated in several different ways: (i) using all available data from respondents at that point in time (i.e. birth and age 7 respectively); (ii) using data from wave 9 respondents only (to assess bias due to non-response at wave 9); and (iii) using HES and/or survey predictors of non-response as auxiliary variables in MI analyses (to assess to what extent sample representativeness can be restored using the selected predictors of non-response).

The second analysis (“Analysis B”) focused on all NCDS cohort members within the wave 9 target population. This includes individuals who did not consent to HES linkage (or who did consent but were ineligible for linkage) and are therefore missing for all HES variables. Analyses related to restoring sample representativeness of early life NCDS variables (father’s social class at birth and cognitive ability at age 7) involved similar comparisons to those outlined for Analysis A. Analyses related to restoring sample representativeness of later life NCDS variables (educational qualifications at age 55 and marital status at age 55) instead considered the percentage without educational qualifications by age 55 and the percentage single and never married by age 55 calculated: (i) among wave 9 respondents; and (ii) using survey or survey and HES predictors of non-response as auxiliary variables in MI analyses. These were then compared to population benchmark values derived from APS.

In each analysis we utilised MI with chained equations [21], generating 20 imputed datasets. All analyses were conducted in Stata 16 and R 4.0.3.

## Results

### HES predictors of NCDS non-response at wave 9 (age 55)

The sample in which we sought to identify HES predictors of NCDS non-response at wave 9 (age 55) was the 6,517 NCDS cohort members who consented to HES linkage at wave 8 (age 50), were considered eligible for HES linkage, and were in the target population at wave 9. Of these, 5,786 (88.8%) responded at wave 9.

Of the 58 HES variables entering the variable selection approach (Supplementary Table S3), 10 were identified as important predictors of NCDS non-response at age 55 (Table 1). Non-response was positively associated with the proportion of OP appointments missed (odds ratio (OR) 1.03 (95% confidence interval (CI) 1.02, 1.03%) comparing those who missed all their appointments vs. those who missed none) and the number of A&E attendances (1.03 (1.01, 1.05) per A&E attendance). Almost all the selected treatments, diagnoses and operations were also positively associated with non-response, with the strongest association being with treatment for adult mental illness (1.73 (1.17, 2.51) for those ever under treatment compared to those never under treatment). The only exception was operation code H (lower digestive tract), where ever having undergone a relevant procedure was found to be protective against non-response (OR 0.73 (95% CI 0.56, 0.93)).

**Table 1:** Estimated odds ratios and 95% confidence intervals for identified Hospital Episode Statistics (HES) predictors of non-response at sweep 9 (age 55) in the 1958 British National Child Development Study (n = 6517)

HES variable^a^	Odds ratio	95% confidence interval
Number of A&E attendances (per unit increase)	1.03	1.01, 1.05
Proportion of OP appointments missed (per unit increase)	1.03	1.02, 1.03
Treatment by Adult Mental Illness	1.73	1.17, 2.51
ICD Chapter IV: Endocrine, nutritional and metabolic diseases	1.17	0.91, 1.51
ICD Chapter V: Mental and behavioural disorders	1.13	0.83, 1.53
ICD Chapter VI: Diseases of the nervous system	1.14	0.85, 1.51
ICD Chapter X: Diseases of the respiratory system	1.20	0.93, 1.53
ICD Chapter XVIII: Symptoms, signs and abnormal clinical and laboratory findings, not elsewhere classified	1.20	0.98, 1.45
Operation code H: Lower digestive tract	0.73	0.56, 0.93
Operation code T: Soft tissue	1.21	0.95, 1.53

Hosmer-Lemeshow goodness-of-fit test p-value = 0.48

Included variables are those identified from application of the least absolute shrinkage and selection operator (LASSO). Results presented here are from a re-estimated multivariable logistic regression model

^a^ Unless otherwise noted, the reference category is having not been diagnosed or treated for the relevant condition

A&E: accident and emergency; OP: outpatients

### Restoring sample representativeness

There was strong evidence of associations between virtually all the identified HES predictors of non-response and cognitive ability at age 7 and no educational qualifications at age 55 (Supplementary Table S4). Evidence of associations with father in professional social class at birth was a little more mixed, while for single and never married at age 55 there was only evidence of association for two of the HES variables. Whilst these results suggest that this set of HES variables may not all be useful auxiliary variables for all the analysis variables, we retained them in the subsequent analyses for completeness. There was strong evidence of association between almost all the identified survey predictors of non-response and the analysis variables (Supplementary Table S5), suggesting that these represent useful auxiliary variables for the intended analyses.

#### Analysis A: HES linkage consenters

**Fig. 2:**
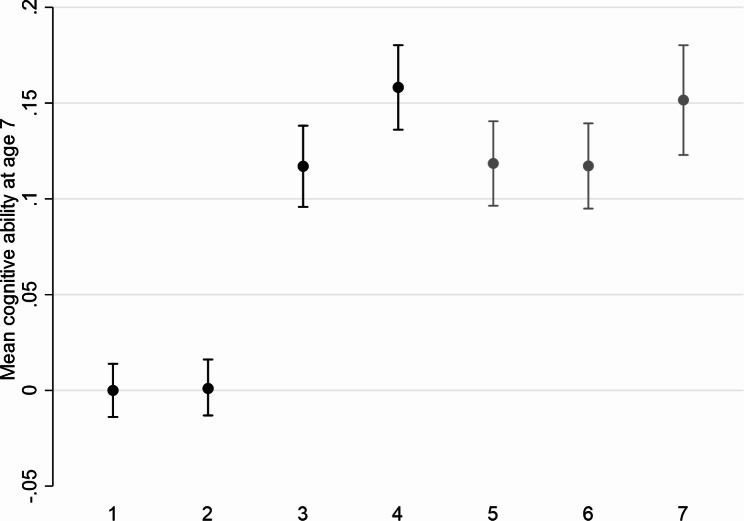
Mean (95% confidence interval) cognitive ability at age 7 in the National Child Development Study before and after handling missing data (Analysis A) Analysis 1: Distribution using all available data (n = 14,407) Analysis 2: Distribution restricted to wave 9 target population (alive and still living in Great Britain) (n = 12,938) Analysis 3: Distribution restricted to wave 9 target population and HES linkage consenters who were eligible for linkage (lived in England for at least one wave been W6 and W9) (n = 5,546) Analysis 4: Distribution restricted to wave 9 respondents within HES linkage consenters who were eligible for linkage (n = 4,928) Analysis 5: MI analysis using only selected survey predictors of non-response as auxiliary variables, restricted to wave 9 target population, HES linkage consenters who were eligible for linkage and those non-missing for the variable of interest, using information on the variable of interest from wave 9 respondents only (n = 5,546) Analysis 6: MI analysis using selected HES predictors of non-response in addition to selected survey predictors of non-response as auxiliary variables, restricted to wave 9 target population, HES linkage consenters who were eligible for linkage and those non-missing for the variable of interest, using information on the variable of interest from wave 9 respondents only (n = 5,546) Analysis 7: MI analysis using only selected HES predictors of non-response as auxiliary variables, restricted to wave 9 target population, HES linkage consenters who were eligible for linkage and those non-missing for the variable of interest, using information on the variable of interest from wave 9 respondents only (n = 5,546)

When restricting to HES linkage consenters who were eligible for linkage, cognitive ability at age 7 had mean 0.12 (95% CI 0.10, 0.14) (Fig. 2). Restricting to wave 9 respondents increased the estimate to 0.16 (95% CI 0.14, 0.18), illustrating substantial bias due to non-response. MI including only the survey predictors of non-response as auxiliary variables reduced the estimate to a level similar to that among all HES linkage consenters who were eligible for linkage (0.12; 95% CI 0.10, 0.14). Additionally including the HES predictors of non-response did not appreciably improve the estimate. Using only the HES predictors of non-response as auxiliary variables had limited impact on restoring sample representativeness (0.15; 95% CI 0.12, 0.18).

Similar findings were observed for father in professional social class at birth (Supplementary Results S1).

#### Analysis B: All NCDS cohort members

**Fig. 3:**
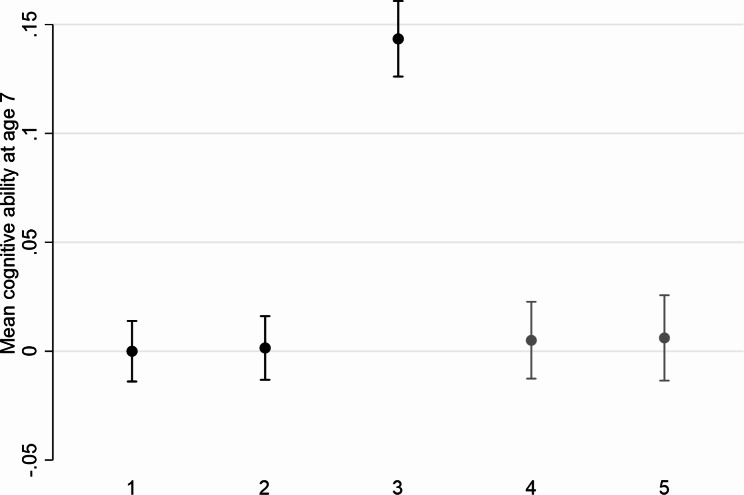
Mean (95% confidence interval) cognitive ability at age 7 in the National Child Development Study before and after handling missing data (Analysis B) Analysis 1: Distribution using all available data (n = 14,407) Analysis 2: Distribution restricted to wave 9 target population (alive and still living in Great Britain) (n = 12,938) Analysis 3: Distribution restricted to wave 9 respondents (n = 7,839) Analysis 4: MI analysis using only selected survey predictors of non-response as auxiliary variables, restricted to wave 9 target population and those non-missing for the variable of interest, using information on the variable of interest from wave 9 respondents only (n = 12,938) Analysis 5: MI analysis using selected HES predictors of non-response in addition to selected survey predictors of non-response as auxiliary variables, restricted to wave 9 target population and those non-missing for the variable of interest, using information on the variable of interest from wave 9 respondents only (n = 12,938)

Cognitive ability at age 7 had mean 0.00 (95% CI -0.01, 0.02) within the wave 9 target population (Fig. 3). When restricting to wave 9 respondents, there was substantial bias (0.14; 95% CI 0.13, 0.16). The MI approach using either only survey predictors of non-response (0.01; 95% CI -0.01, 0.02) or both survey and HES predictors of non-response (0.01; 95% CI -0.01, 0.03) successfully overcame this bias and restored sample representativeness.

The percentage of NCDS-comparable individuals in the population without educational qualifications was estimated to be 12.3% (95% CI 10.9%, 13.8%) using APS data (Fig. S4). Using NCDS wave 9 respondents, this was instead estimated to be 8.4% (95% CI 7.9%, 9.0%), demonstrating considerable bias relative to the population benchmark. MI estimates using survey predictors of non-response (13.7%; 95% CI 12.8%, 14.6%) or survey and HES predictors of non-response (13.7%; 95% CI 12.7%, 14.6%) were much closer to the population estimates (and with point estimates inside the population 95% CI).

Similar findings were observed for father in professional social class at birth and single and never married at age 55 (Supplementary Results S1).

## Discussion

### Summary of findings

Our analysis identified 10 HES variables associated with NCDS age 55 non-response. Most of the identified variables signified poor health, either through A&E attendances or through diagnosis of or treatment for a specific disease or condition. Whilst the existing literature on predictors of non-response in longitudinal surveys has not generally examined this area in such detail, our observations are consistent with previous findings that worse physical [2, 22–24] and mental [24–27] health are associated with subsequent non-response. There is also potential overlap with previously identified survey predictors of NCDS age 55 non-response such as self-rated general health in mid-life and conduct problems in adolescence [12]. Our work in identifying HES predictors of subsequent NCDS non-response provides evidence of potential medical reasons for wave non-response, though this is an inherently partial view of the overall reasons for non-participation.

There was generally strong evidence that the identified HES predictors of non-response were associated with the variables considered in the analyses looking to restore sample representativeness, suggesting that they may constitute useful auxiliary variables. Whilst the inclusion of HES predictors of non-response as auxiliary variables did aid in restoring sample representative to a limited extent, in analyses where the previously identified survey predictors of non-response were used there was generally no benefit of additionally including the HES variables. These results are suggestive that, for these specific variables at least, the survey predictors of non-response were sufficient to fulfil the MAR assumption, with the HES variables largely superfluous in this regard.

### Strengths and limitations

There are several strengths to this analysis. This study used a large, long-running, population-representative cohort study. We utilised a data-driven approach, eschewing a theory-based approach to allow us to identify variables which may aid in maximising the plausibility of the MAR assumption without preconceptions. We explored the performance of our proposed approach to the handling of missing data through comparison to population benchmarks.

However, there are also a number of limitations. Our restriction to higher level derived diagnosis and treatment HES variables may mean that more granular relevant information was overlooked. Our finding that there was essentially no additional gain in sample representativeness when using HES predictors of NCDS wave 9 non-response relative to analyses using only previously identified survey predictors of non-response may be specific to this setting. In other cohorts and/or linked administrative datasets, variables derived from the administrative data which are predictive of non-response may potentially be of greater value. In particular, NCDS already contains a very rich set of socio-economic and health variables, which may reduce the potential for added value from other data sources – this may not be the case in other cohorts. Linked administrative data may also provide useful auxiliary variables on the basis of their association with the underlying values of variable(s) subject to missingness, for example by acting as a proxy for a partially observed outcome variable [28, 29]. This needs to be addressed on an analysis-specific basis so has not been considered here but is an important area for future work.

More broadly, a potential limitation of using linked administrative data in the handling of missing data lies in the nature of the linkage consent mechanism. In NCDS, opt-in linkage consent was sought at wave 8 (age 50), meaning that any cohort members who did not respond at this wave (including anyone who attritted prior to this point) will not have linked HES data – yet these individuals will constitute a large proportion of non-respondents at subsequent waves, so are a subgroup for whom appropriate non-response handling is essential. This emphasises the importance of early (ideally at study initiation) opt-in linkage consents or alternative (e.g. opt-out) consent mechanisms to allow access to linked data for as many study participants as possible. It also highlights the potential of surveys which utilise an administrative data sampling frame, meaning that some administrative data should be available for all sampled individuals, including baseline non-participants, allowing particularly thorough investigation of non-response.

### Implications for analyses using NCDS data

We have demonstrated that principled methods for missing data handling (in this case MI) utilising appropriately chosen auxiliary variables have the ability to restore sample representativeness in NCDS. Whilst the inclusion of HES predictors of non-response did aid in restoring sample representative to a limited extent, previously identified survey predictors of non-response were far more important. For users of NCDS data, we therefore emphasise previous guidance on the inclusion of appropriately chosen survey predictors of non-response in analyses [12, 30] and do not suggest the default inclusion of HES variables on the basis of their association with non-response. However, as noted, auxiliary variables should also be considered based on their association with the underlying values of variable(s) subject to missingness, and HES variables may therefore be relevant for certain analyses.

## Conclusions

In this analysis we explored the extent to which administrative (HES) data could aid in predicting survey (NCDS) non-response and restoring survey sample representativeness. Whilst the inclusion of HES predictors of non-response did aid in restoring sample representative to a limited extent, previously identified survey predictors of non-response were the far more important, highlighting their value in analyses of data subject to missingness. However, these findings may not generalise to other analyses or cohorts. This work provides a demonstration of the use of linked administrative data for the handling of missing cohort data which we hope will act as template for others.

### Electronic supplementary material

Below is the link to the electronic supplementary material.

Supplementary Material 1

## Data Availability

The datasets analysed during the current study are from a publicly available source, the UK Data Service [https://beta.ukdataservice.ac.uk/datacatalogue/series/series?id=2000032].

## List of abbreviations

A&E: Accident and Emergency
APC: Admitted Patient Care
APS: Annual Population Survey
CC: Critical Care
FIML: Full information maximum likelihood
HES: Hospital Episode Statistics
IPW: Inverse probability weighting
LASSO: Least absolute shrinkage and selection operator
MAR: Missing at random
MI: Multiple imputation
NCDS: 1958 National Child Development Study
ONS: Office for National Statistics
OP: Outpatient
