# Supplementary material for: Using linked administrative data to aid the handling of non-response and restore sample representativeness in cohort studies: the 1958 national child development study and hospital episode statistics data
Source: BMC Med Res Methodol. 2023 Nov 11;23:266. doi: 10.1186/s12874-023-02099-w (PMC10638694; doi:10.1186/s12874-023-02099-w)
Supplement: Supplementary file 1 — Supplementary Material 1 [file 12874_2023_2099_MOESM1_ESM.docx]

**SUPPLEMENTARY MATERIAL**

Using linked administrative data to aid the handling of non-response and restore sample representativeness in cohort studies: the 1958 National Child Development Study and Hospital Episode Statistics data

Nasir Rajah^1^

Lisa Calderwood^1^

Bianca L De Stavola^2^

Katie Harron^2^

George B Ploubidis^1^

Richard J Silverwood^1^*

1 Centre for Longitudinal Studies, UCL Social Research Institute, University College London, London, UK

2 Population, Policy & Practice Research and Teaching Department, UCL Great Ormond Street Institute of Child Health, University College London, London, UK

*Corresponding author:

Richard J Silverwood

[R.Silverwood@ucl.ac.uk](mailto:R.Silverwood@ucl.ac.uk)

**Contents**

[**Methods S1**. Secondary variable selection analysis. 3](#_Toc149217192)

[**Methods S2**. Sample representativeness analyses. 5](#_Toc149217193)

[**Results S1**. Sample representativeness analyses. 9](#_Toc149217194)

[**Table S1**. Survey-based predictors of National Child Development Study non-response at wave 9 (age 55). 10](#_Toc149217195)

[**Table S2**. Derivation of Hospital Episode Statistics variables for consideration as potential predictors of National Child Development Study non-response. 11](#_Toc149217196)

[**Table S3**. Descriptive statistics for Hospital Episode Statistics (HES) variables used in the analysis. 13](#_Toc149217197)

[**Table S4**. Associations between analysis variables and HES predictors of non-response at sweep 9 (age 55) in the 1958 British National Child Development Study (NCDS). 18](#_Toc149217198)

[**Table S5**. Associations between analysis variables and survey predictors of non-response at sweep 9 (age 55) in the 1958 British National Child Development Study (NCDS). 19](#_Toc149217199)

[**Figure S1**. Flow diagram showing 1958 British National Child Development Study-Hospital Episode Statistics data linkage and data availability. 21](#_Toc149217200)

[**Figure S2.** Percentage (95% confidence interval) of fathers in professional social class at birth in the National Child Development Study before and after handling missing data (Analysis A). 23](#_Toc149217201)

[**Figure S3**. Percentage (95% confidence interval) of fathers in professional social class at birth in the National Child Development Study before and after handling missing data (Analysis B). 24](#_Toc149217202)

[**Figure S4**. Percentage (95% confidence interval) of cohort members without any educational qualifications at age 55 in the National Child Development Study before and after handling missing data (Analysis B). 25](#_Toc149217203)

[**Figure S5**. Percentage (95% confidence interval) of cohort members who are single and never married at age 55 in the National Child Development Study before and after handling missing data (Analysis B). 26](#_Toc149217204)

## **Methods S1**. Secondary variable selection analysis.

Methods

In a secondary analysis we used a multi-stage p-value-based variable selection approach, similar to that employed by Mostafa et al (1), for comparison with the primary approach using the LASSO. We employed a modified Poisson model with robust standard errors (2) to estimate risk ratios at each stage:

Stage 1: Univariable modified Poisson regressions of non-response at age 55 on each identified predictor from the HES datasets. Retain variables where P < 0.05.

Stage 2: Multivariable modified Poisson regression of non-response at age 55 on all predictors retained from stage 1. Retain variables where P < 0.05

In the interests of transparency, we note that this approach was initially planned to be our primary analysis with the LASSO a secondary analysis, but we have switched their reporting due to poor performance (in terms of number of predictors of non-response identified) of the p-value-based approach. As the ultimate aim of the present analysis was to identify a set of variables containing sufficient information with respect to NCDS age 55 non-response to act as useful auxiliary variables in subsequent analyses, we considered this change in methodological focus to be necessary and do not believe that it adversely affects the interpretation of our findings.

Results

Fifty-eight variables were derived from HES data and formed the input to stage 1 of the process to identify predictors of non-response at age 55. In the secondary analysis, 22 of these were found to have univariable associations with non-response, so passed from stage 1 to stage 2. In stage 2, three of these variables remained associated with non-response in the multivariable model so were identified as important HES predictors of non-response:

1. Proportion of outpatient appointments missed
2. Number of A&E attendances
3. Treatment for adult mental illness (ever treated)

Since only a small number of predictors of non-response was identified using this approach, and since they were a subset of those identified in the primary analysis using LASSO, these variables were not considered further in isolation.

**References**

1. Mostafa T, Narayanan M, Pongiglione B, Dodgeon B, Goodman A, Silverwood RJ, et al. Missing at random assumption made more plausible: evidence from the 1958 British birth cohort. J Clin Epidemiol. 2021;136:44-54.

2. Zou G. A modified poisson regression approach to prospective studies with binary data. Am J Epidemiol. 2004;159(7):702-6.

## **Methods S2**. Sample representativeness analyses.

We undertook a number of different analyses to assess how effective the identified HES predictors of NCDS age 55 non-response were at restoring sample representativeness despite selective attrition. We considered sample representativeness in terms of two NCDS variables observed in early life (father’s social class at birth and cognitive ability at age 7) and in terms of two NCDS variables observed in later life (educational qualifications at wave 9 (age 55) and marital status at wave 9 (age 55)). We undertook two different analyses to explore different aspects of restoring sample representativeness, the first (“Analysis A”) considering only HES linkage consenters and the second (“Analysis B”) considering all NCDS cohort members. All four NCDS variables were considered within Analysis B, but only the two early life variables within Analysis A. Full details of the analyses are provided below.

**Analysis A: HES linkage consenters**

This analysis focused on HES linkage consenters who were eligible for linkage (lived in England for at least one wave been wave 6 and wave 9) and who were within the wave 9 target population. These individuals are non-missing for all HES variables, therefore it is possible to conduct MI analyses where the only auxiliary variables are the selected HES predictors of non-response, allowing an assessment of whether the HES variables in isolation are helpful in restoring sample representativeness. However, analyses restricted to HES linkage consenters form only a limited proportion of analyses that could be undertaken using NCDS data and results in this setting may not be more broadly applicable. As these analyses relate to a subpopulation which would be impossible to identify within the APS (individuals who would hypothetically consent to HES linkage), these analyses were restricted to restoring sample representativeness of variables observed in early life (father’s social class at birth and cognitive ability at age 7).

*Restoring sample representativeness of NCDS variables observed in early life (father’s social class at birth and cognitive ability at age 7): wave 9 respondents only vs. complete distribution from early life*

Analyses:

1. Distribution using all available data
2. Distribution restricted to wave 9 target population (alive and still living in GB)
3. Distribution restricted to wave 9 target population and HES linkage consenters who were eligible for linkage (lived in England for at least one wave been W6 and W9)
4. Distribution restricted to wave 9 respondents within HES linkage consenters who were eligible for linkage
5. MI analysis using only selected survey predictors of non-response as auxiliary variables, restricted to wave 9 target population, HES linkage consenters who were eligible for linkage and those non-missing for the variable of interest, using information on the variable of interest from wave 9 respondents only
6. MI analysis using selected HES predictors of non-response in addition to selected survey predictors of non-response as auxiliary variables, restricted to wave 9 target population, HES linkage consenters who were eligible for linkage and those non-missing for the variable of interest, using information on the variable of interest from wave 9 respondents only
7. MI analysis using only selected HES predictors of non-response as auxiliary variables, restricted to wave 9 target population, HES linkage consenters who were eligible for linkage and those non-missing for the variable of interest, using information on the variable of interest from wave 9 respondents only

Comparisons of interest:

- 2 vs. 1 shows any difference between wave 9 target population and all available data
- 3 vs 2 shows any bias introduced by only considering HES linkage consenters who were eligible for linkage (among the wave 9 target population)
- 4 vs. 3 shows any bias due to non-response at wave 9 (among the wave 9 target population and HES linkage consenters who were eligible for linkage)
- 5 vs. 4 shows to what extent sample representativeness can be restored using only the selected survey predictors of non-response (among the wave 9 target population and HES linkage consenters who were eligible for linkage)
- 6 vs. 4 shows to what extent sample representativeness can be restored using both the selected HES predictors of non-response and the selected survey predictors of non-response (among the wave 9 target population and HES linkage consenters who were eligible for linkage)
- 6 vs. 5 shows the added value of the selected HES predictors of non-response relative to only the selected survey predictors of non-response for restoring sample representativeness (among the wave 9 target population and HES linkage consenters who were eligible for linkage)
- 7 vs. 4 shows to what extent sample representativeness can be restored using only the selected HES predictors of non-response (among the wave 9 target population and HES linkage consenters who were eligible for linkage)

**Analysis B: All NCDS cohort members**

This analysis focused on all NCDS cohort members (within the wave 9 target population). This includes individuals who did not consent to HES linkage (or who did consent but were ineligible for linkage) and are therefore missing for all HES variables, making it impossible to conduct MI analyses where the only auxiliary variables are the selected HES predictors of non-response, meaning an assessment of whether the HES variables in isolation are helpful in restoring sample representativeness is not possible. However, analyses of all NCDS cohort members will be more commonly undertaken than those restricted to HES linkage consenters who were eligible for linkage (Analysis A) so there is much interest in restoring sample representativeness in this setting. As these analyses relate to the whole NCDS sample (which should be representative of the population), corresponding population statistics can be obtained from APS to serve as population benchmarks. Analyses related to restoring sample representativeness of both early life (father’s social class at birth and cognitive ability at age 7) and later life (educational qualifications at wave 9 (age 55) and marital status at wave 9 (age 55)) NCDS variables were undertaken, though these were by necessity structured slightly differently.

*Restoring sample representativeness of NCDS variables observed in early life (father’s social class at birth and cognitive ability at age 7): wave 9 respondents only vs. complete distribution from early life*

Analyses:

1. Distribution using all available data
2. Distribution restricted to wave 9 target population (alive and still living in GB)
3. Distribution restricted to wave 9 respondents
4. MI analysis using only selected survey predictors of non-response as auxiliary variables, restricted to wave 9 target population and those non-missing for the variable of interest, using information on the variable of interest from wave 9 respondents only
5. MI analysis using selected HES predictors of non-response in addition to selected survey predictors of non-response as auxiliary variables, restricted to wave 9 target population and those non-missing for the variable of interest, using information on the variable of interest from wave 9 respondents only

Comparisons of interest:

- 2 vs. 1 shows any difference between wave 9 target population and all available data
- 3 vs. 2 shows any bias due to non-response at wave 9 (among the wave 9 target population)
- 4 vs. 3 shows to what extent sample representativeness can be restored using only the selected survey predictors of non-response (among the wave 9 target population)
- 5 vs. 3 shows to what extent sample representativeness can be restored using both the selected HES variables and the selected survey predictors of non-response (among the wave 9 target population)
- 5 vs. 4 shows the added value of the selected HES predictors of non-response relative to only the selected survey predictors of non-response for restoring sample representativeness (among the wave 9 target population)

*Restoring sample representativeness of NCDS variables observed in later life (marital status at wave 9 (age 55) and educational qualifications at wave 9 (age 55)): wave 9 respondents vs. population benchmark data from APS*

Analyses:

1. Distribution using population benchmark data from APS
2. Distribution among wave 9 respondents (which in this case is all available information)
3. MI analysis using only selected survey predictors of non-response as auxiliary variables, restricted to wave 9 target population
4. MI analysis using selected HES predictors of non-response in addition to selected survey predictors of non-response as auxiliary variables, restricted to wave 9 target population

Comparisons of interest:

- 2 vs. 1 shows any bias introduced by selective response at wave 9 respondents (among the wave 9 target population)
- 3 vs. 2 shows to what extent sample representativeness can be restored using only the selected survey predictors of non-response
- 4 vs. 2 shows to what extent sample representativeness can be restored using both the selected HES variables and the selected survey predictors of non-response
- 4 vs. 3 shows the added value of the selected HES predictors of non-response relative to only the selected survey predictors of non-response for restoring sample representativeness

## **Results S1**. Sample representativeness analyses.

**Analysis A: HES linkage consenters**

Using all available data, 4.5% of cohort members had a father in the professional social class (95% CI 4.2%, 4.9%) (Fig. S2), with a similar estimate within the wave 9 target population. Amongst the HES linkage consenters who were eligible for linkage the estimate increased slightly to 5.0% (95% CI 4.5%, 5.6%), demonstrating moderate bias due to selection into linkage consent. When considering only wave 9 respondents the estimate was 5.3% (95% CI 4.7%, 6.0%), showing limited bias due to non-response. None of the MI approaches overcame this limited non-response bias, with similar estimates when using only survey predictors of non-response (5.4%; 95% CI 4.8%, 6.0%), both survey and HES predictors of non-response (5.4%; 95% CI 4.8%, 6.0%), or only HES predictors of non-response (5.3%; 95% CI 4.7%, 6.0%).

**Analysis B: All NCDS cohort members**

The proportion of cohort members whose father was in the professional social class was 4.5% (95% CI 4.2%, 4.9%) using all available data (Fig. S3), with a similar distribution when restricting to the wave 9 target population (4.6%; 95% CI 4.2%, 4.9%). There was evidence of bias (non-overlapping CIs) when restricting to wave 9 respondents (5.4%; 95% CI 5.0%, 6.0%), though this was overcome using MI utilising either the survey predictors of non-response alone (4.7%; 95% CI 4.3%, 5.2%) or in combination with the HES predictors of non-response (4.7%; 95% CI 4.3%, 5.3%).

The APS-derived estimated prevalence of being single and never been married of 11.4% had a relatively wide 95% CI (10.0%, 12.8%) (Fig. S5). The corresponding estimate among NCDS wave 9 respondents 10.0% (95% CI 9.3%, 10.6%), showing relative bias in the point estimate even if there remained overlap in the 95% CIs. The MI analysis using only survey predictors of non-response overcame most of this bias (10.9%; 95% CI 10.2%, 11.7%), though the combination of survey and HES predictors of non-response performed even better (11.5%; 95% CI 10.8%, 12.4%).

## **Table S1**. Survey-based predictors of National Child Development Study non-response at wave 9 (age 55).

| Sweep | Variable |
| --- | --- |
| Sweep 0 (age 0) | Mother’s age |
|  | Number of persons per room |
|  | Parity |
|  | Social class of mother's father when she left school |
|  | Sex of child |
|  | Social class of mother’s husband |
| Sweep 1 (age 7) | Dad stayed on at school after minimum age |
|  | Social problems (alcoholism etc.) |
|  | Cognitive ability summary |
|  | Ever breastfed |
| Sweep 2 (age 11) | Cognitive ability summary |
|  | Conduct problems |
| Sweep 3 (age 16) | Child receiving help at school – backwardness |
|  | Child's school attendance |
|  | How long since child drank alcohol |
|  | Test 2 – mathematics comprehension |
|  | Conduct problems |
| Sweep 4 (age 23) | Legal marital status |
|  | Voted in 1979 general election |
| Sweep 5 (age 33) | Telephone in home |
|  | How much physical effort in job |
|  | Voted in 1987 general election |
|  | Housing tenure |
|  | Social capital score (people turn to for advice, support) |
| Sweep 6 (age 42) | Membership in organisations |
| Biomedical sweep (age 44) | Self-rated general health |
| Sweep 7 (age 46) | Marital status - de facto |
| Sweep 8 (age 50) | Total number of natural children |
|  | Employer provided pension scheme |
|  | Non-response at sweeps 1-8 |

## **Table S2**. Derivation of Hospital Episode Statistics variables for consideration as potential predictors of National Child Development Study non-response.

| Variable | Number of variables | Details | HES dataset | Period |
| --- | --- | --- | --- | --- |
| Number of A&E attendances | 1 | Count of the number of times the individual has had an A&E appointment. | A&E | 2007-2012 |
| Proportion of A&E investigations to attendances | 1 | The individual’s average number of investigations in A&E divided by the number of A&E attendances the individual has. | A&E | 2007-2012 |
| Average number of A&E treatments | 1 | The average number of treatments the individual received in A&E, which is the number of treatments divided by the number of A&E visits. | A&E | 2007-2012 |
| Number of APC spells | 1 | Count of the number of spells an individual has, defined by number of unique admission dates. | APC | 1997-2012 |
| Number of OP appointments | 1 | Count of the number of appointments in the outpatient data. | OP | 2003-2012 |
| Percentage of OP appointments missed | 1 | The number of outpatient appointments missed divided by the total number of outpatient appointments the individual has. Only derived for individuals with a minimum of two outpatient appointments. | OP | 2003-2012 |
| OP treatment (High Dependency Care, Intensive Care, Oncologist, Rehabilitation, Adult Mental Illness, Cardiology, Plastic Surgery) | 7 | Whether the individual had ever been treated under the relevant area at any point; 0 if they had not and 1 if they had. | OP | 2003-2012 |
| Diagnosis codes (A-Z) | 21 | Whether the individual had the diagnosis code (ICD-10) recorded at any point: 0 if they had not, 1 if they had. | APC | 1997-2012 |
| Operation codes (A-Z) | 24 | Whether the individual had the operation code (OPCS-4) recorded at any point: 0 if they had not, 1 if they had. | APC | 1997-2012 |
| **Total** | **58** |  |  |  |

HES: Hospital Episode Statistics; APC: admitted patient care; A&E: accident and emergency; OP: outpatients; ICD-10: International Statistical Classification of Diseases and Related Health Problems 10th Revision; OPCS-4: Office of Population Censuses and Surveys Classification of Interventions and Procedures version 4.

Note: We assume that cohort members who were eligible for and consented to linkage but did not have linked data for a given HES dataset truly did not have a relevant interaction (e.g., admission, outpatient appointment) with an NHS hospital in England over the corresponding time period. Such individuals are therefore included in the analysis with HES variables derived to reflect that, for example, if they had no linked HES APC data then they did not receive any APC-based diagnoses or undergo any APC-based treatments.

## **Table S3**. Descriptive statistics for Hospital Episode Statistics (HES) variables used in the analysis.

|  | Overall  (n = 6517) | Respondents  (n = 5786) | Non-respondents  (n = 731) |
| --- | --- | --- | --- |
| HES variable | Mean (SD) | Mean (SD) | Mean (SD) |
| Number of A&E attendances | 1.5 (3.1) | 1.4 (2.9) | 2.2 (4.1) |
| Proportion of A&E investigations to attendances | 0.3 (0.9) | 0.3 (0.9) | 0.3 (0.8) |
| Average number of A&E treatments | 0.6 (1.2) | 0.6 (1.2) | 0.7 (1.1) |
| Number of APC spells | 2.4 (4.4) | 2.3 (4.1) | 3.1 (6.0) |
| Number of OP appointments | 11.1 (18.6) | 10.8 (17.7) | 13.9 (24.4) |
| Percentage of OP appointments missed | 3.9 (9.2) | 3.5 (8.5) | 7.2 (13.0) |
| HES variable | n (% of total) | n (% of those in HES variable stratum) | n (% of those in HES variable stratum) |
| Treatment by High Dependency Care |  |  |  |
| No | 6466 (99.2%) | 5742 (88.8%) | 724 (11.2%) |
| Yes | 51 (0.8%) | 44 (86.3%) | 7 (13.7%) |
| Treatment by Intensive Care |  |  |  |
| No | 6498 (99.7%) | 5769 (88.8%) | 729 (11.2%) |
| Yes | 19 (0.3%) | 17 (89.5%) | 2 (10.5%) |
| Treatment by Oncologist |  |  |  |
| No | 6440 (98.8%) | 5719 (88.8%) | 721 (11.2%) |
| Yes | 77 (1.2%) | 67 (87.0%) | 10 (13.0%) |
| Treatment by Rehabilitation |  |  |  |
| No | 6450 (99.0%) | 5729 (88.8%) | 721 (11.2%) |
| Yes | 67 (1.0%) | 57 (85.1%) | 10 (14.9%) |
| Treatment by Adult Mental Illness |  |  |  |
| No | 6337 (97.2%) | 5652 (89.2%) | 685 (10.8%) |
| Yes | 180 (2.8%) | 134 (74.4%) | 46 (25.6%) |
| Treatment by Cardiology |  |  |  |
| No | 5829 (99.4%) | 5196 (89.1%) | 633 (10.9%) |
| Yes | 688 (10.6%) | 590 (85.8%) | 98 (14.2%) |
| Treatment by Plastic Surgery |  |  |  |
| No | 6259 (96.0%) | 5561 (88.8%) | 698 (11.2%) |
| Yes | 258 (4.0%) | 225 (87.2%) | 33 (12.8%) |
| ICD Chapter I: Certain infectious and parasitic diseases |  |  |  |
| No | 6254 (96.0%) | 5566 (89.0%) | 688 (11.0%) |
| Yes | 263 (4.0%) | 220 (83.7%) | 43 (16.3%) |
| ICD Chapter II: Neoplasms |  |  |  |
| No | 5940 (91.1%) | 5279 (88.9%) | 661 (11.1%) |
| Yes | 577 (8.9%) | 507 (87.9%) | 70 (12.1%) |
| ICD Chapter III: Diseases of the blood and blood-forming organs and certain disorders involving the immune mechanism |  |  |  |
| No | 6323 (97.0%) | 5618 (88.9%) | 705 (11.1%) |
| Yes | 194 (3.0%) | 168 (86.6%) | 26 (13.4%) |
| ICD Chapter IV: Endocrine, nutritional and metabolic diseases |  |  |  |
| No | 5894 (90.4%) | 5266 (89.3%) | 628 (10.7%) |
| Yes | 623 (9.6%) | 520 (83.5%) | 103 (16.5%) |
| ICD Chapter V: Mental and behavioural disorders |  |  |  |
| No | 6100 (93.6%) | 5448 (89.3%) | 652 (10.7%) |
| Yes | 417 (6.4%) | 338 (81.1%) | 79 (18.9%) |
| ICD Chapter VI: Diseases of the nervous system |  |  |  |
| No | 6064 (93.0%) | 5406 (89.1%) | 658 (10.9%) |
| Yes | 453 (7.0%) | 380 (83.9%) | 73 (16.1%) |
| ICD Chapter VII: Diseases of the eye and adnexa |  |  |  |
| No | 6296 (96.6%) | 5599 (88.9%) | 697 (11.1%) |
| Yes | 221 (3.4%) | 187 (84.6%) | 34 (15.4%) |
| ICD Chapter VIII: Diseases of the ear and mastoid process |  |  |  |
| No | 6425 (98.6%) | 5709 (88.9%) | 716 (11.1%) |
| Yes | 92 (1.4%) | 77 (83.7%) | 15 (16.3%) |
| ICD Chapter IX: Diseases of the circulatory system |  |  |  |
| No | 5412 (83.0%) | 4816 (89%) | 596 (11%) |
| Yes | 1105 (17.0%) | 970 (87.8%) | 135 (12.2%) |
| ICD Chapter X: Diseases of the respiratory system |  |  |  |
| No | 5865 (90.0%) | 5240 (89.3%) | 625 (10.7%) |
| Yes | 652 (10.0%) | 546 (83.7%) | 106 (16.3%) |
| ICD Chapter XI: Diseases of the digestive system |  |  |  |
| No | 4956 (76.0%) | 4412 (89.0%) | 544 (11.0%) |
| Yes | 1561 (24.0%) | 1374 (88.0%) | 187 (12.0%) |
| ICD Chapter XII: Diseases of the skin and subcutaneous tissue |  |  |  |
| No | 6091 (93.5%) | 5404 (88.7%) | 687 (11.3%) |
| Yes | 426 (6.5%) | 382 (89.7%) | 44 (10.3%) |
| ICD Chapter XIII: Diseases of the musculoskeletal system and connective tissue |  |  |  |
| No | 5352 (82.1%) | 4782 (89.3%) | 570 (10.7%) |
| Yes | 1165 (17.9%) | 1004 (86.2%) | 161 (13.8%) |
| ICD Chapter XIV: Diseases of the genitourinary system |  |  |  |
| No | 5322 (81.7%) | 4739 (89%) | 583 (11%) |
| Yes | 1195 (18.3%) | 1047 (87.6%) | 148 (12.4%) |
| ICD Chapter XV: Pregnancy, childbirth and the puerperium |  |  |  |
| No | 6336 (97.2%) | 5625 (88.8%) | 711 (11.2%) |
| Yes | 181 (2.8%) | 161 (89%) | 20 (11%) |
| ICD Chapter XVII: Congenital malformations, deformations and chromosomal abnormalities |  |  |  |
| No | 6504 (99.8%) | 5773 (88.8%) | 731 (11.2%) |
| Yes | 13 (0.2%) | 13 (100%) | 0 (0%) |
| ICD Chapter XVIII: Symptoms, signs and abnormal clinical and laboratory findings, not elsewhere classified |  |  |  |
| No | 4998 (76.7%) | 4489 (89.8%) | 509 (10.2%) |
| Yes | 1519 (23.3%) | 1297 (85.4%) | 222 (14.6%) |
| ICD Chapter XIX: Injury, poisoning and certain other consequences of external causes |  |  |  |
| No | 5819 (89.3%) | 5195 (89.3%) | 624 (10.7%) |
| Yes | 698 (10.7%) | 591 (84.7%) | 107 (15.3%) |
| ICD Chapter XX: External causes of morbidity and mortality |  |  |  |
| No | 6073 (93.2%) | 5415 (89.2%) | 658 (10.8%) |
| Yes | 444 (6.8%) | 371 (83.6%) | 73 (16.4%) |
| ICD Chapter XXI: Factors influencing health status and contact with health services |  |  |  |
| No | 4658 (71.5%) | 4154 (89.2%) | 504 (10.8%) |
| Yes | 1859 (28.5%) | 1632 (87.8%) | 227 (12.2%) |
| ICD Chapter XXII: Codes for special purposes |  |  |  |
| No | 6512 (99.9%) | 5782 (88.8%) | 730 (11.2%) |
| Yes | 5 (0.1%) | 4 (80%) | 1 (20%) |
| Operation code A – Nervous system |  |  |  |
| No | 6130 (94.1%) | 5449 (88.9%) | 681 (11.1%) |
| Yes | 387 (5.9%) | 337 (87.1%) | 50 (12.9%) |
| Operation code B – Endocrine system |  |  |  |
| No | 6325 (97.0%) | 5616 (88.8%) | 709 (11.2%) |
| Yes | 192 (3.0%) | 170 (88.5%) | 22 (11.5%) |
| Operation code C - Eye |  |  |  |
| No | 6306 (96.8%) | 5606 (88.9%) | 700 (11.1%) |
| Yes | 211 (3.2%) | 180 (85.3%) | 31 (14.7%) |
| Operation code D – Ear |  |  |  |
| No | 6492 (99.6%) | 5764 (88.8%) | 728 (11.2%) |
| Yes | 25 (0.4%) | 22 (88.0%) | 3 (12.0%) |
| Operation code E – Respiratory tract |  |  |  |
| No | 6214 (95.4%) | 5525 (88.9%) | 689 (11.1%) |
| Yes | 303 (4.6%) | 261 (86.1%) | 42 (13.9%) |
| Operation Code F – Mouth |  |  |  |
| No | 6216 (95.4%) | 5521 (88.8%) | 695 (11.2%) |
| Yes | 301 (4.6%) | 265 (88.0%) | 36 (12.0%) |
| Operation code G – Upper digestive tract |  |  |  |
| No | 5805 (89.1%) | 5161 (88.9%) | 644 (11.1%) |
| Yes | 712 (10.9%) | 625 (87.8%) | 87 (12.2%) |
| Operation code H – Lower digestive tract |  |  |  |
| No | 5684 (87.2%) | 5042 (88.7%) | 642 (11.3%) |
| Yes | 833 (12.8%) | 744 (89.3%) | 89 (10.7%) |
| Operation code J – Other abdominal organs (principally digestive) |  |  |  |
| No | 6348 (97.4%) | 5641 (88.9%) | 707 (11.1%) |
| Yes | 169 (2.6%) | 145 (85.8%) | 24 (14.2%) |
| Operation code K – Heart |  |  |  |
| No | 6292 (96.5%) | 5597 (89.0%) | 695 (11.0%) |
| Yes | 225 (3.5%) | 189 (84.0%) | 36 (16.0%) |
| Operation code L – Arteries and veins |  |  |  |
| No | 6291 (96.5%) | 5586 (88.8%) | 705 (11.2%) |
| Yes | 226 (3.5%) | 200 (88.5%) | 26 (11.5%) |
| Operation code M – Urinary |  |  |  |
| No | 6075 (93.2%) | 5408 (89%) | 667 (11%) |
| Yes | 442 (6.8%) | 378 (85.5%) | 64 (14.5%) |
| Operation code N – Male genital organs |  |  |  |
| No | 6318 (96.9%) | 5603 (88.7%) | 715 (11.3%) |
| Yes | 199 (3.1%) | 183 (92%) | 16 (8%) |
| Operation code P – Lower female genital tract |  |  |  |
| No | 6357 (97.5%) | 5645 (88.8%) | 712 (11.2%) |
| Yes | 160 (2.5%) | 141 (88.1%) | 19 (11.9%) |
| Operation code Q – Upper female genital tract |  |  |  |
| No | 5699 (87.4%) | 5052 (88.6%) | 647 (11.4%) |
| Yes | 818 (12.6%) | 734 (89.7%) | 84 (10.3%) |
| Operation code R – Female genital tract |  |  |  |
| No | 6378 (97.9%) | 5660 (88.7%) | 718 (11.3%) |
| Yes | 139 (2.1%) | 126 (90.6%) | 13 (9.4%) |
| Operation code S - Skin |  |  |  |
| No | 5966 (91.5%) | 5309 (89.0%) | 657 (11.0%) |
| Yes | 551 (8.5%) | 477 (86.6%) | 74 (13.4%) |
| Operation code T – Soft tissue |  |  |  |
| No | 5827 (89.4%) | 5198 (89.2%) | 629 (10.8%) |
| Yes | 690 (10.6%) | 588 (85.2%) | 102 (14.8%) |
| Operation code U – Diagnostic imaging, testing and rehabilitation |  |  |  |
| No | 6145 (94.3%) | 5470 (89.0%) | 675 (11.0%) |
| Yes | 372 (5.7%) | 316 (84.9%) | 56 (15.1%) |
| Operation code V – Bones and joints of skull and spine |  |  |  |
| No | 6358 (97.6%) | 5647 (88.8%) | 711 (11.2%) |
| Yes | 159 (2.4%) | 139 (87.4%) | 20 (12.6%) |
| Operation code W – Other bones and joints |  |  |  |
| No | 5736 (88.0%) | 5101 (88.9%) | 635 (11.1%) |
| Yes | 781 (12.0%) | 685 (87.7%) | 96 (12.3%) |
| Operation code X – Miscellaneous operation |  |  |  |
| No | 6157 (94.5%) | 5480 (89.0%) | 677 (11.0%) |
| Yes | 360 (5.5%) | 306 (85.0%) | 54 (15.0%) |
| Operation code Y – Methods of operation not elsewhere classifiable |  |  |  |
| No | 5085 (78.0%) | 4552 (89.5%) | 533 (10.5%) |
| Yes | 1432 (22.0%) | 1234 (86.2%) | 198 (13.8%) |
| Operation code Z – Subsidiary classification |  |  |  |
| No | 3864 (59.3%) | 3460 (89.5%) | 404 (10.5%) |
| Yes | 2653 (40.7%) | 2326 (87.7%) | 327 (12.3%) |

SD: Standard Deviation; APC: admitted patient care; CC: critical care; A&E: accident and emergency; OP: outpatients.

## **Table S4**. Associations between analysis variables and HES predictors of non-response at sweep 9 (age 55) in the 1958 British National Child Development Study (NCDS).

|  | HES predictors of NCDS age 55 non-response | | | | | | | | | |
| --- | --- | --- | --- | --- | --- | --- | --- | --- | --- | --- |
|  | 1 | 2 | 3 | 4 | 5 | 6 | 7 | 8 | 9 | 10 |
| Father in professional social class at birth | 0.01 | 0.02 | 0.06 | 0.001 | 0.10 | 0.54 | 0.16 | 0.06 | 0.72 | 0.07 |
| Cognitive ability at age 7 | <0.001 | <0.001 | 0.001 | <0.001 | <0.001 | <0.001 | <0.001 | <0.001 | <0.001 | <0.001 |
| No educational qualifications at age 55 | <0.001 | <0.001 | <0.001 | <0.001 | <0.001 | <0.001 | <0.001 | <0.001 | 0.21 | 0.04 |
| Single and never married at age 55 | 0.25 | 0.28 | 0.008 | 0.73 | 0.004 | 0.63 | 0.92 | 0.33 | 0.61 | 0.80 |

p-values from Wald tests of parameter(s) in logistic regression models. Colour coding corresponds to the magnitude of the p-value, from green (0) to red (1).

1. Number of A&E attendances
2. Proportion of OP appointments missed
3. Treatment by Adult Mental Illness
4. ICD Chapter IV: Endocrine, nutritional and metabolic diseases
5. ICD Chapter V: Mental and behavioural disorders
6. ICD Chapter VI: Diseases of the nervous system
7. ICD Chapter X: Diseases of the respiratory system
8. ICD Chapter XVIII: Symptoms, signs and abnormal clinical and laboratory findings, not elsewhere classified
9. Operation code H: Lower digestive tract
10. Operation code T: Soft tissue

## **Table S5**. Associations between analysis variables and survey predictors of non-response at sweep 9 (age 55) in the 1958 British National Child Development Study (NCDS).

|  | Survey predictors of NCDS age 55 non-response | | | | | | | | | | | | | | |
| --- | --- | --- | --- | --- | --- | --- | --- | --- | --- | --- | --- | --- | --- | --- | --- |
|  | 1 | 2 | 3 | 4 | 5 | 6 | 7 | 8 | 9 | 10 | 11 | 12 | 13 | 14 | 15 |
| Father in professional social class at birth | <0.001 | <0.001 | <0.001 | <0.001 | 0.93 |  | <0.001 | <0.001 | <0.001 | <0.001 | <0.001 | 0.001 | <0.001 | <0.001 | 0.004 |
| Cognitive ability at age 7 | <0.001 | <0.001 | <0.001 | <0.001 | <0.001 | <0.001 | <0.001 | <0.001 |  | <0.001 | <0.001 | <0.001 | <0.001 | <0.001 | <0.001 |
| No educational qualifications at age 55 | 0.37 | <0.001 | <0.001 | <0.001 | 0.74 | <0.001 | <0.001 | <0.001 | <0.001 | <0.001 | <0.001 | <0.001 | <0.001 | <0.001 | <0.001 |
| Single and never married at age 55 | <0.001 | 0.67 | 0.41 | 0.12 | <0.001 | 0.001 | 0.01 | 0.86 | 0.01 | 0.58 | 0.08 | 0.13 | <0.001 | 0.85 | <0.001 |
|  | Survey predictors of NCDS age 55 non-response | | | | | | | | | | | | | | |
|  | 16 | 17 | 18 | 19 | 20 | 21 | 22 | 23 | 24 | 25 | 26 | 27 | 28 | 29 | 30 |
| Father in professional social class at birth | <0.001 | <0.001 | <0.001 | <0.001 | <0.001 | <0.001 | 0.58 | <0.001 | 0.06 | <0.001 | <0.001 | 0.03 | 0.35 | 0.04 | 0.001 |
| Cognitive ability at age 7 | <0.001 | <0.001 | <0.001 | <0.001 | <0.001 | <0.001 | <0.001 | <0.001 | <0.001 | <0.001 | <0.001 | <0.001 | 0.01 | <0.001 | <0.001 |
| No educational qualifications at age 55 | <0.001 | <0.001 | <0.001 | <0.001 | <0.001 | <0.001 | <0.001 | <0.001 | <0.001 | <0.001 | <0.001 | 0.001 | 0.002 | <0.001 | <0.001 |
| Single and never married at age 55 | 0.55 | 0.12 | <0.001 | 0.71 | <0.001 | 0.75 | 0.15 | <0.001 | <0.001 | 0.01 | <0.001 | <0.001 | <0.001 | 0.11 | <0.001 |

p-values from Wald tests of parameter(s) in logistic regression models. Colour coding corresponds to the magnitude of the p-value, from green (0) to red (1).

1. Mother’s age (Sweep 0)
2. Number of persons per room (Sweep 0)
3. Parity (Sweep 0)
4. Social class of mother's father when she left school (Sweep 0)
5. Sex of child (Sweep 0)
6. Social class of mother’s husband (Sweep 0)
7. Dad stayed on at school after minimum age (Sweep 1)
8. Social problems (alcoholism etc.) (Sweep 1)
9. Cognitive ability summary (Sweep 1)
10. Ever breastfed (Sweep 1)
11. Cognitive ability summary (Sweep 2)
12. Conduct problems (Sweep 2)
13. Child receiving help at school – backwardness (Sweep 3)
14. Child's school attendance (Sweep 3)
15. How long since child drank alcohol (Sweep 3)
16. Test 2 – mathematics comprehension (Sweep 3)
17. Conduct problems (Sweep 3)
18. Legal marital status (Sweep 4)
19. Voted in 1979 general election (Sweep 4)
20. Telephone in home (Sweep 5)
21. How much physical effort in job (Sweep 5)
22. Voted in 1987 general election (Sweep 5)
23. Housing tenure (Sweep 5)
24. Social capital score (people turn to for advice, support) (Sweep 5)
25. Membership in organisations (Sweep 6)
26. Self-rated general health (Biomedical sweep)
27. Marital status - de facto (Sweep 7)
28. Total number of natural children (Sweep 8)
29. Employer provided pension scheme (Sweep 8)
30. Non-response at sweeps 1-8

Note: Results omitted in cases where the analysis variable is derived from (or is identical to) the survey predictor of NCDS age 55 non-response.

**
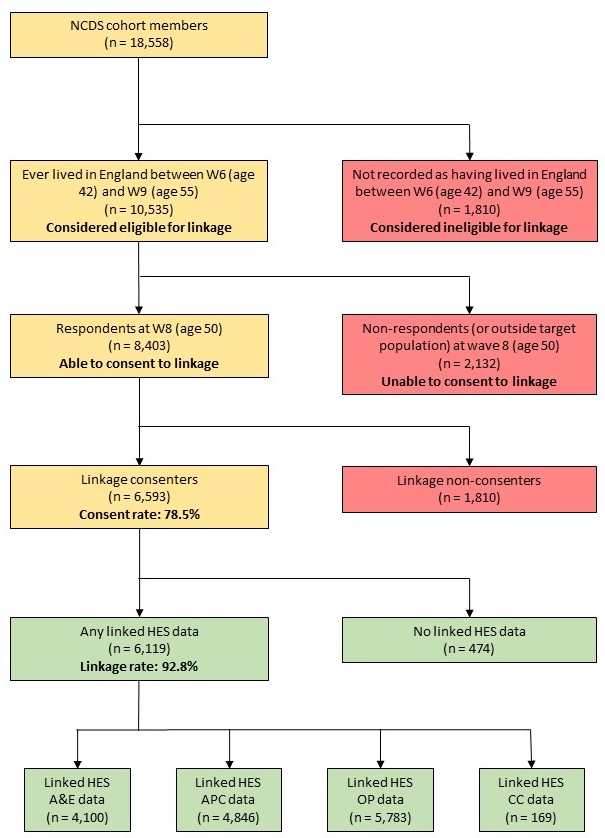
**

## **Figure S1**. Flow diagram showing 1958 British National Child Development Study-Hospital Episode Statistics data linkage and data availability.

We consider cohort members to be eligible for linkage with HES data if they reported living in England at any one or more waves between waves 6 (2000, age 42) and 9 (2013, age 55), corresponding to the period of HES data availability (1997 onwards). Of the 10,535 cohort members meeting this definition of linkage eligibility, 8,403 (79.8%) responded at wave 8, with 6,593 (78.5% of respondents) providing consent for linkage. Among these linkage consenters, 6,119 had linked data from one or more of the HES datasets, giving a linkage rate of 92.8%.

APC: admitted patient care; CC: critical care; A&E: accident and emergency; OP: outpatients.

## **Figure S2.** Percentage (95% confidence interval) of fathers in professional social class at birth in the National Child Development Study before and after handling missing data (Analysis A).

Analysis 1: Distribution using all available data (n = 16,458).

Analysis 2: Distribution restricted to wave 9 target population (alive and still living in GB) (n = 13,880).

Analysis 3: Distribution restricted to wave 9 target population and HES linkage consenters who were eligible for linkage (lived in England for at least one wave been W6 and W9) (n = 5,867).

Analysis 4: Distribution restricted to wave 9 respondents within HES linkage consenters who were eligible for linkage (n = 5,226).

Analysis 5: MI analysis using selected survey predictors of non-response as auxiliary variables, restricted to wave 9 target population, HES linkage consenters who were eligible for linkage and those non-missing for the variable of interest, using information on the variable of interest from wave 9 respondents only (n = 5,867).

Analysis 6: MI analysis using both selected HES predictors of non-response and selected survey predictors of non-response as auxiliary variables, restricted to wave 9 target population, HES linkage consenters who were eligible for linkage and those non-missing for the variable of interest, using information on the variable of interest from wave 9 respondents only (n = 5,867).

Analysis 7: MI analysis using selected HES predictors of non-response as auxiliary variables, restricted to wave 9 target population, HES linkage consenters who were eligible for linkage and those non-missing for the variable of interest, using information on the variable of interest from wave 9 respondents only (n = 5,867).

## **Figure S3**. Percentage (95% confidence interval) of fathers in professional social class at birth in the National Child Development Study before and after handling missing data (Analysis B).

Analysis 1: Distribution using all available data (n = 16,458).

Analysis 2: Distribution restricted to wave 9 target population (alive and still living in GB) (n = 13,880).

Analysis 3: Distribution restricted to wave 9 respondents (n = 8,284).

Analysis 4: MI analysis using selected survey predictors of non-response as auxiliary variables, restricted to wave 9 target population and those non-missing for the variable of interest, using information on the variable of interest from wave 9 respondents only (n = 13,880).

Analysis 5: MI analysis using both selected HES predictors of non-response and selected survey predictors of non-response as auxiliary variables, restricted to wave 9 target population and those non-missing for the variable of interest, using information on the variable of interest from wave 9 respondents only (n = 13,880).

## **Figure S4**. Percentage (95% confidence interval) of cohort members without any educational qualifications at age 55 in the National Child Development Study before and after handling missing data (Analysis B).

Analysis 1: Distribution using population benchmark data from APS (n = 1,935).

Analysis 2: Distribution among wave 9 respondents (which in this case is all available information) (n = 8,952).

Analysis 3: MI analysis using selected survey predictors of non-response as auxiliary variables, restricted to wave 9 target population (n = 15,613).

Analysis 4: MI analysis using both selected HES predictors of non-response and selected survey predictors of non-response as auxiliary variables, restricted to wave 9 target population (n = 15,613).

## **Figure S5**. Percentage (95% confidence interval) of cohort members who are single and never married at age 55 in the National Child Development Study before and after handling missing data (Analysis B).

Analysis 1: Distribution using population benchmark data from APS (n = 1,937).

Analysis 2: Distribution among wave 9 respondents (which in this case is all available information) (n = 9,130).

Analysis 3: MI analysis using selected survey predictors of non-response as auxiliary variables, restricted to wave 9 target population (n = 15,613).

Analysis 4: MI analysis using both selected HES predictors of non-response and selected survey predictors of non-response as auxiliary variables, restricted to wave 9 target population (n = 15,613).
